# A Novel Conflict Management Method Based on Uncertainty of Evidence and Reinforcement Learning for Multi-Sensor Information Fusion

**DOI:** 10.3390/e23091222

**Published:** 2021-09-17

**Authors:** Fanghui Huang, Yu Zhang, Ziqing Wang, Xinyang Deng

**Affiliations:** School of Electronics and Information, Northwestern Polytechnical University, Xi’an 710072, China; huangfanghui@mail.nwpu.edu.cn (F.H.); zhangyuchn@mail.nwpu.edu.cn (Y.Z.); wangziqing@mail.nwpu.edu.cn (Z.W.)

**Keywords:** multi-sensor information fusion, negation of evidence, reinforcement learning, uncertainty degree, correlation coefficient

## Abstract

Dempster–Shafer theory (DST), which is widely used in information fusion, can process uncertain information without prior information; however, when the evidence to combine is highly conflicting, it may lead to counter-intuitive results. Moreover, the existing methods are not strong enough to process real-time and online conflicting evidence. In order to solve the above problems, a novel information fusion method is proposed in this paper. The proposed method combines the uncertainty of evidence and reinforcement learning (RL). Specifically, we consider two uncertainty degrees: the uncertainty of the original basic probability assignment (BPA) and the uncertainty of its negation. Then, Deng entropy is used to measure the uncertainty of BPAs. Two uncertainty degrees are considered as the condition of measuring information quality. Then, the adaptive conflict processing is performed by RL and the combination two uncertainty degrees. The next step is to compute Dempster’s combination rule (DCR) to achieve multi-sensor information fusion. Finally, a decision scheme based on correlation coefficient is used to make the decision. The proposed method not only realizes adaptive conflict evidence management, but also improves the accuracy of multi-sensor information fusion and reduces information loss. Numerical examples verify the effectiveness of the proposed method.

## 1. Introduction

Multi-sensor information fusion (MSIF) is an important information processing technology, which can achieve multi-level and multi-source information combination optimization [1,2]. A single sensor has less information and is easily affected by environmental interference and measurement error. As a result, the obtained information may contain mistakes, which makes it difficult to make accurate decisions [3]. In contrast, fusing multi-sensor information can improve the performance of system and make the results more reliable [4,5]. Due to the advantages of multi-sensor setups, in recent years, it has been widely used in fault diagnosis, target positioning, and UAV system control [6,7,8,9,10]. The practical experience shows that comparing with a single-sensor system, multi-sensor systems can significantly enhance the system performance of detection, identification, and fault diagnosis [11,12]; however, due to various uncertainties in the real world, the information obtained by multi-sensor is affected. In addition, due to the influence of the sensor itself, the information obtained by multi-sensor systems may be inaccurate, uncertain, or even be faulty [13,14,15]. How to correctly process multi-sensor information and establish a fusion model is a widespread attention problem. As for this issue, many theories and methods have been proposed, for example Z-number [16,17], D-number [18,19], fuzzy sets [20,21,22], rough sets [23,24], R-number [25], entropy-based [26,27], and Dempster–Shafer theory (DST) [28,29].

DST is an uncertainty reasoning theory, as an extension of probability theory, which can process uncertain information without prior probability [29]. Due to the characteristics, DST has been widely used in military and civil fields. In addition, DST provides a classic combination rule for fusing multi-source information, namely Dempster’s combination rule (DCR); however, DCR has some problems in application. When the evidence to combine is highly conflicting, it may produce counter-intuitive results, for example, the Zadeh paradox [30]. Facing with these challenges, many methods have been proposed in the past years. Yager [31] considered that the conflict cannot provide useful information. He proposed a combination rule that redistributes the conflict to the frame of discernment (FOD). Dubois and Prade [32] proposed that the conflict should be assigned to the intersection or union of associated focal elements. Later, Murphy [33] proposed that the original evidence should be given weights for modification and to obtain new evidence. Then, the new evidence was used to achieve multi-sensor information fusion (MSIF) based on the DCR. Lefevre et al. [34] proposed a general framework to realize the unification of several classical combination rules. Smets [35] thought the conflict should be allocated to empty set. Dezert and Smarandache [36] proposed a new framework i.e., Dezert–Smarandache Theory (DSmT), which is an extension of DST. Further, in [36], a series of combined rules are provided, namely PCR1-PCR6, which can handle conflicting evidence. Based on interval-valued belief structures, Song et al. [37] presented an uncertainty measurement method and applied the method to MSIF. Aiming at the fusion decision making without prior knowledge, Wang et al. [38] designed a method based on interval-valued belief structure and DCR. Yuan and Xiao et al. [39] proposed a fusion method based on Deng entropy [40] and evidence distance [41]. Jiang and Wei et al. [42] proposed a weighted average method based on the credibility of evidence to deal with high-conflict evidence. Ni et al. [43] presented an improved conflict evidence fusion method, in which the degree of uncertainty of evidence was used to design the weight coefficient of each evidence.

The above methods mainly focus on original basic probability assignment (BPA); however the concept of negative evidence is also a feasible way to express information. Through the negation, multi-faceted aspects of information can be viewed. Smets proposed a calculation method for determining the negation of probabilistic events [44]. Based on that, many scholars have carried out relevant research on the negation of BPA, and proposed a series of approaches for the negation of BPA [45,46,47,48]. In addition, researchers adopt different methods to measure the uncertainty of BPA, and modified the original BPA based on the uncertainty for the combination of evidence.

Until now, the above-mentioned methods cannot realize the real-time conflict processing and the calculation is complicated when the amount of data is large. This paper proposes a new information fusion method, which combines the uncertainty of evidence and RL. In the proposed method, the negation of evidence is calculated. Then, Deng entropy is used to measure the uncertainty of evidence. Moreover, in order to avoid the irrationality caused by the conflict of information, RL is used to realize adaptive conflict resolution of evidence. Finally, DCR and correlation coefficient are used for multi-sensor information fusion and decision making. In the proposed method, we consider the original BPA and the negation of BPA, the reason is as follows. The positive information of the evidence can be obtained from the original BPA, the negative information of the evidence can be obtained from the negative BPA. Through the original BPA and negation of BPA can make the information obtained more comprehensive.

The main contributions are summarized as follows:

- The negation of evidence is introduced into RL to achieve information quality assessment. The uncertainty of original evidence and its negation is obtained by using Deng entropy. Then, the obtained uncertainty degrees are used to distinguish the information quality of evidence, which helps to realize the access to information.
- In order to achieve the adaptive online information fusion, RL is combined with the uncertainty degrees to process the conflicting evidence. In this process, a Markov decision process (MDP) model is built, and solved through Q-learning algorithm to implement the fusion of evidence.

The rest of this paper is organized as follows. In Section 2, the preliminaries, including DST, the negation of BPA, Deng entropy, and RL are introduced. In Section 3, the proposed information fusion decision method is presented. In Section 4, the effectiveness the proposed method is verified by numerical examples. Finally, in Section 5, the conclusion is given.

## 2. Preliminaries

### 2.1. Dempster–Shafer Theory (DST)

DST is an effective method to deal with uncertain information, which satisfies weaker conditions than Bayesian probability [29]. Some basic concepts in DST are given below.

Assume Θ is a finite set consisting of *N* mutually exclusive elements, indicated by

(1) $$\Theta \;=\;\left\{\theta_{1},\theta_{2},\ldots ,\theta_{N}\right\},$$

then the Θ is called a FOD.

The power set of Θ is indicated by

(2) $$2^{\Theta }=\left\{\theta_{1},\theta_{2},\cdots ,\theta_{N},\left\{\theta_{1},\theta_{2}\right\},\left\{\theta_{1},\theta_{3}\right\},\cdots ,\Theta ,\emptyset \right\}.$$

If a function $m:2^{\Theta }\to \left[0,1\right]$ satisfies the following conditions, it is a BPA or mass function,

(3) $$\left\{\begin{array}{c} m(\emptyset )=0\\ \sum m(A)=1 \end{array}\right.$$

where *A* is called focal element, and m(A) represents the mass assigned to *A*.

DST provides a Dempster’s combination rule (DCR) [28,29] to fuse multiple pieces of evidence, which is defined as below

(4) $$\left\{\begin{array}{c} m(\emptyset )=0\\ m\left(A\right)=\frac{\sum_{A_{1}\cap A_{2}\cap A_{3}\cdots =A}m_{1}\left(A_{1}\right)m_{2}\left(A_{2}\right)\cdots m_{m}\left(A_{m}\right)}{1-K}\left(A\neq \emptyset \right), \end{array}\right.$$

where $K=\sum_{A_{1}\cap A_{2}\cap A_{3}\cdots =\emptyset }m_{1}\left(A_{1}\right)m_{2}\left(A_{2}\right)\cdots m_{m}\left(A_{m}\right)$ represents the conflict among BPAs.

Yager’s combination rule [31] is an alternative for the combination of evidence, which is defined as below

(5) $$\left\{\begin{array}{c} m\left(A\right)=\sum_{A_{1}\cap A_{2}=A}m_{1}\left(A_{1}\right)m_{2}\left(A_{2}\right),\left(A\neq \emptyset ,\Theta \right)\\ m\left(\Theta \right)=\sum_{A_{1}\cap A_{2}=\Theta }m_{1}\left(A_{1}\right)m_{2}\left(A_{2}\right)+k\\ m(\emptyset )=0, \end{array}\right.$$

where $k=\sum_{A_{1}\cap A_{2}=\emptyset }m_{1}\left(A_{1}\right)m_{2}\left(A_{2}\right)$.

### 2.2. Negation of Evidence

The negation is an important way to express information. Recently, Deng and Jiang [45] proposed a BPA negation calculation method based on maximum uncertainty allocation.

Given a FOD Θ, for each focal element $A_{i}$, assuming $m\left(A_{i}\right)=\alpha_{i}$, the negation of *m* is denoted as $\bar{m}$:

(1) If $A_{i}$ is a singleton θ, then $\bar{m}\left({\bar{A}}_{i}\right)=\alpha_{i}$, where ${\bar{A}}_{i}=\Theta -A_{i}$;

(2) If $A_{i}$ is not a singleton, then $\bar{m}\left({\bar{A}}_{i}\right)=\alpha_{i}$, where ${\bar{A}}_{i}=\cup_{\forall \theta \in A_{i}}\left(\Theta -\theta \right)$.

It can be seen from the above that, for an evidence *m*, the negation of *m* can be calculated by

(6) $$\bar{m}\left(B\right)=\sum_{A_{i}satisfying\left(\underset{\forall \theta \in A_{i}}{\cup }\left(\Theta -\theta \right)\right)=B}m(A_{i}),$$

where B⊆Θ.

### 2.3. Deng Entropy

Deng entropy [40] is a method to calculate the uncertainty of evidence, and it is an extension of Shannon entropy [49]. The specific definition of Deng entropy is given as follows

(7) $$E_{d}=-\sum_{i}m\left(A_{i}\right)\log\frac{m(A_{i})}{2^{\left|A_{i}\right|}-1},$$

where $\left|A_{i}\right|$ is the cardinality of *A*.

When dealing with a bayesian BPA, Deng entropy degenerates to Shannon entropy, which is

(8) $$E_{d}=-\sum_{i}m\left(A_{i}\right)\log\frac{m(A_{i})}{2^{\left|A_{i}\right|}-1}=-\sum_{i}m(A_{i})\log m\left(A_{i}\right).$$

### 2.4. Correlation Coefficient

For a FOD with *N* elements, assuming that there are two BPAs are $m_{1}$ and $m_{2}$, respectively, then the correlation coefficient between $m_{1}$ and $m_{2}$ is defined as follows [50]

(9) $$r_{BPA}\left(m_{1},m_{2}\right)=\frac{c(m_{1},m_{2})}{\sqrt{c\left(m_{1},m_{1}\right)\times c\left(m_{2},m_{2}\right)}},$$

where $c(m_{1},m_{2})$ is defined as

(10) $$c\left(m_{1},m_{2}\right)=\sum_{i=1}^{2^{N}}\sum_{j=1}^{2^{N}}m_{1}\left(A_{i}\right)m_{2}\left(A_{j}\right)\frac{\left|A_{i}\cap A_{j}\right|}{\left|A_{i}\cup A_{j}\right|},$$

where |·| is the cardinality of a set.

The correlation coefficient $r_{BPA}\left(m_{1},m_{2}\right)$ indicates the correlation between $m_{1}$ and $m_{2}$. The larger the correlation coefficient, the higher the degree of correlation between $m_{1}$ and $m_{2}$.

### 2.5. Reinforcement Learning (RL)

RL does not require any data to be given in advance, which obtains the reward by the continuous interaction between agent and environment. By employing the RL, a system dynamically adjusts the parameters to maximize the accumulated reward [51,52]. In RL, the return function is usually defined to represent the sum of the discounts of all rewards observed by the agent after a certain state, i.e.,

(11) $$G_{t}=\left(R_{t+1}+\gamma R_{t+2}+\gamma^{2}R_{t+3}+\cdots \right)=\sum_{k=0}^{\infty }R_{t+k+1},$$

where, γ is the discount factor (γ∈[0,1)), which represents the weight relationship between future rewards and immediate reward, and *R* is the immediate reward.

In RL, the value function is used to evaluate the expected return in a certain state, which do not consider the actions taken at this time, only consider the current system state, and defined as

(12) $$\begin{array}{c} V\left(s\right)=E_{\pi }\left(G_{t}|S_{t}=s\right)\\ \;\;\;\;\;=E_{\pi }\left(R_{t+1}+\gamma R_{t+2}+\gamma^{2}R_{t+3}+\cdots |S_{t}=s\right). \end{array}$$

The Bellman equation of value function is given as follows

(13) $$V\left(s\right)=E_{\pi }\left(R_{t}+\gamma v_{\pi }\left(s^{\prime }\right)|S_{t}=s\right).$$

$V^{*}\left(s\right)$ is the optimal value function, i.e.,

(14) $$V^{*}\left(s\right)=E_{\pi }\left(R_{t}+\gamma v^{*}\left(s^{\prime }\right)|S_{t}=s\right).$$

Since V(s) cannot evaluate the impact of a certain action on the system, a state-action value function (Q value function) is proposed. Q value function is used to evaluate the expected return in a certain policy. The policy is defined as π:S→A, defined as $\pi \left(a|s\right)=P\left(A_{t}=a|S_{t}=s\right)$. In other word, Q value function is the expectation of the cumulative reward obtained when the agent in state *s* adopts action *a*, which is defined as

(15) $$\begin{array}{c} Q_{\pi }\left(s,a\right)=E_{\pi }\left(G_{t}|S_{t}=s,A_{t}=a\right)\\ \;\;\;\;=E_{\pi }\left(R_{t+1}+\gamma R_{t+2}+\gamma^{2}R_{t+3}+\cdots |S_{t}=s,A_{t}=a\right). \end{array}$$

The Bellman equation of Q value function is given as follows

(16) $$Q_{\pi }\left(s,a\right)=E_{\pi }\left(R_{t+1}+\gamma Q_{\pi }\left(S_{t+1},A_{t+1}\right)|S_{t}=s,A_{t}=a\right).$$

$Q^{*}\left(s,a\right)$ is the optimal Q value function, i.e.,

(17) $$Q^{*}\left(s,a\right)=E_{\pi }\left(R_{t+1}+\gamma Q^{*}\left(S_{t+1},A_{t+1}\right)|S_{t}=s,A_{t}=a\right).$$

We can obtain the optimal policy from $V^{*}\left(s\right)$ and $Q^{*}\left(s,a\right)$.

(18) $$\pi^{*}=\arg\max_{a\in A}V^{*}\left(s\right)=\arg\max_{a\in A}Q^{*}\left(s,a\right).$$

## 3. The Proposed Method

**Figure 1:**
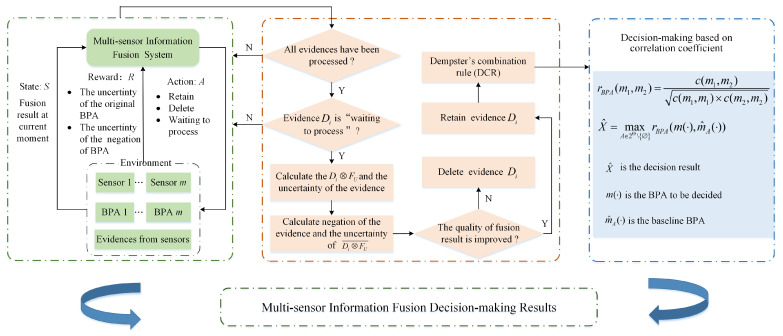
The overall framework of the proposed method.

In this section, a novel evidence combination method is proposed for adapting conflict and making fusion decisions based on the uncertainty of evidence and RL. This method defines information fusion as a RL task, and builds a fusion model using RL and the uncertainty of original BPA and are calculated by the use of Deng entropy comprehensively. Firstly, considering that the negation of BPA is also an important way to express information, the uncertainty of original BPA and its negation. If we adopt the negation of BPA and the original BPA as the judgment conditions. Then the judgment conditions are diversified, which can help to obtain the correct processing results of different sensor information and realize effective conflict management. If we adopt the original BPA as the judgment condition. Then the judgment condition is single, which may cause inaccurate processing results of the sensor information. Thus, these two uncertainty degrees as the judgment conditions are used to distinguish the information quality of evidence, so that consistent evidence can be selected through RL. Next DCR is used to implement information fusion. Finally, the decision result is obtained through a decision-making scheme based on correlation coefficients. The overall information fusion and decision process of the proposed method is shown in Figure 1.

### 3.1. Markov Decision Process (MDP)

In the fusion decision system, the next state is obtained by selecting an action under the current system state. A MDP is built for the multi-sensor information fusion decision system.

#### 3.1.1. Action Set

Due to the impact of the actual environment, the multi-sensor information fusion decision system may be of high conflict; therefore, it needs to set up a reasonable action policy to realize the effective processing of conflicting data. In our proposed method, the action set *A* is defined as

(19) $$A=\left\{a_{1},a_{2},a_{3}\right\}=\{\mathrm{Retain},\mathrm{Delete},\mathrm{Waiting}\;\;\mathrm{to}\;\;\mathrm{process}\}.$$

An evidence can be retained through action $a_{1}$, whose information can be fused later. A high-conflict evidence can be deleted through action $a_{2}$, which can avoid the adverse impact of conflicting evidence on fusion results. An evidence with a low degree of conflict or with a small amount of information can be temporarily retained through action $a_{3}$, i.e., “waiting to process”. A “waiting to process” evidence will be operated in the subsequent steps. After the first round of screening of all the evidence, the evidence of “waiting to process” will process again. Specifically, all the evidence retained in the first round is fused and denoted as $F_{U}$. Then the evidence of “waiting to process” will be reconsidered until the uncertainty of evidence obtained by combination is satisfied.

#### 3.1.2. State Set

In RL, when an action is taken, the state of the system will change in another state. In the fusion system, when the system action changes, the fusion result changes. Thus, we define the current fused result as the system state, i.e.,

(20) $$s_{t+1}=m_{t+1}=\left\{\begin{array}{c} m_{t}⊕D_{t+1},a_{t+1}=\mathrm{Retain}\\ \;\;\;m_{t},\;\;a_{t+1}=\mathrm{Delete}\\ \;\;\;m_{t},\;\;a_{t+1}=\mathrm{Waiting}\;\;\mathrm{to}\;\;\mathrm{process}, \end{array}\right.$$

where $m_{t}$ represents the fusion result at time *t*, $D_{t+1}$ is the sensor evidence at time t+1, and $a_{t+1}$ represents the action taken at time t+1.

Based on the above analysis, the system state set can be defined as

(21) $$S=\{s_{1},s_{2},\cdots ,s_{t},s_{t+1},\cdots \}.$$

#### 3.1.3. Reward

Reward is a feedback value given by the environment in a certain state *s* and certain action *a*. In this paper, the environment is mainly containing the sensor information and the fusion result at each time. The system uses reward value to determine the optimal action at each time. In this paper, there are two cases. Case 1: The evidence is not in conflict, then the fusion of evidence will generate consistent results. Case 2: The evidence is in conflict, then the quality of fusion result is not guaranteed. In this paper, we use Deng entropy to evaluate the quality of fusion results so as to set the reward function. The reason is as follows.

According in Equation (7), Deng entropy uses $m\left(A\right)\log\left(2^{\left|A\right|}-1\right)$ to represent nonspecificity, which not only contains focal elements, but represents the power set of FOD. Deng entropy is more sensitive to the change of focal elements. When the focal element changes, the uncertainty of BPA also changes strongly. In RL, we use the uncertainty of BPA to make policy for sensor information. The stronger the uncertainty, the stronger the feedback signal for RL, the more conducive RL to make accurate policy.

The uncertainty of the original BPA is defined as E(m). At the same time Deng entropy is also adopted to calculate the uncertainty of the negation of *m*, defined as $E(\bar{m})$. These two uncertainties are denoted as

(22) $$\left\{\begin{array}{c} E\left(m\right)=-\sum_{A\in 2^{\Theta }}m\left(A\right)\log(\frac{m(A)}{2^{\left|A\right|}-1})\\ E\left(\bar{m}\right)=-\sum_{A\in 2^{\Theta }}\bar{m}\left(A\right)\log(\frac{\bar{m}\left(A\right)}{2^{\left|A\right|}-1}) \end{array}\right.$$

Then E(m) and $E(\bar{m})$ are jointly used to judge the quality of information. Specifically, it can be divided into the following cases.

Case 1: If $\left\{\begin{array}{c} E\left(m_{t+1}\right)\leq E\left(m_{t}\right)\\ E\left({\bar{m}}_{t+1}\right)\leq E\left({\bar{m}}_{t}\right) \end{array}\right.$, it indicates that the new state $s_{t+1}$ is with less uncertainty from both positive and negative view of information, which should be given a positive reward, since adding new evidence leads to more certain fusion result.

Case 2: If $\left\{\begin{array}{c} E\left(m_{t+1}\right)>E\left(m_{t}\right)\\ E\left({\bar{m}}_{t+1}\right)>E\left({\bar{m}}_{t}\right) \end{array}\right.$, it indicates that the new state $s_{t+1}$ is with larger uncertainty from both positive and negative view of information, which should be given a penalty reward, since adding new evidence leads to more uncertain fusion result.

Case 3: If $\left\{\begin{array}{c} E\left(m_{t+1}\right)<E\left(m_{t}\right)\\ E\left({\bar{m}}_{t+1}\right)>E\left({\bar{m}}_{t}\right) \end{array}\right.$ or $\left\{\begin{array}{c} E\left(m_{t+1}\right)>E\left(m_{t}\right)\\ E\left({\bar{m}}_{t+1}\right)<E\left({\bar{m}}_{t}\right) \end{array}\right.$, it indicates that the effect of the new state $s_{t+1}$ cannot be determined, which will not be rewarded or penalized. Therefore, the evidence in this case is waiting to be processed.

By setting the above three cases, we can adopt different policies for sensors (i.e., delete, retain, or waiting to process), so as to delete the high conflict evidence and retain the valid evidence.

Given the above analysis the reward function in this paper is defined as

(23) $$R_{t+1}=\left\{\begin{array}{c} 20,\;E\left(m_{t+1}\right)\leq E\left(m_{t}\right)\&E\left({\bar{m}}_{t+1}\right)\leq E\left({\bar{m}}_{t}\right)\\ 0,\;\;\;E\left(m_{t+1}\right)>E\left(m_{t}\right)\&E\left({\bar{m}}_{t+1}\right)<E\left({\bar{m}}_{t}\right)\\ 0,\;\;\;E\left(m_{t+1}\right)<E\left(m_{t}\right)\&E\left({\bar{m}}_{t+1}\right)>E\left({\bar{m}}_{t}\right)\\ -20,\;\;E\left(m_{t+1}\right)>E\left(m_{t}\right)\&E\left({\bar{m}}_{t+1}\right)>E\left({\bar{m}}_{t}\right) \end{array}\right.$$

### 3.2. Q-Learning Algorithm Solution

After modeling the MDP, we adopt a model-free Q-learning algorithm to obtain the optimal policy [53]. The main reasons are as shown as follows.

Reason 1: The system in this paper is a discrete system, and Q-learning is suitable for a discrete system.

Reason 2: The state-action space is small in this system. Hence the system does not require a neural network to store state-action.

Reason 3: The state transition probability of the system is unknown, so a model-free algorithm is needed.

Q-learning is used to find high-quality evidence by removing deletion of conflicting BPAs, which is the main idea of obtaining the optimal fusion result. Specifically, at time *t*, the system receives BPAs from different sensors, then it uses the action selection policy to select an action $a_{t}$. Herein, a ε−greedy policy is utilized to select the action, which is to explore new actions with a probability of ε, and select optimal action currently considered with a probability of 1−ε. The ε−greedy policy can ensure the balance between the exploration and exploitation of the algorithm. The specific definition is as follows.

(24) $$\pi^{*}\left(a|s\right)=\left\{\begin{array}{c} 1-\varepsilon +\frac{\varepsilon }{m},\mathrm{if}\;\;a=\arg\max Q\left(s,a\right)\\ \frac{\varepsilon }{m},\;\;\;\;\;\mathrm{if}\;\;a\neq \arg\max Q\left(s,a\right), \end{array}\right.$$

where *m* represents all optional actions, and Q(s,a) represents the Q value of the Q value function in state *s* and action *a*.

Then, the fusion system performs action $a_{t}$ and obtains a new fusion result (i.e, a new BPA). At time *t*, the uncertainty of original BPA and the negative BPA is measured by Deng entropy, and compared with the uncertainty at time t−1. A reward value at time *t* is obtained according to the reward function. Equation (25) is used to calculate the current Q value, and the Q value is stored in the Q table. We have

(25) $$Q\left(s_{t},a_{t}\right)=R\left(s_{t},a_{t}\right)+\sum_{t=1}^{+\infty }\gamma^{t}R\left(s_{t},a_{t}\right),$$

where γ is the discount factor.

The fusion system selects actions according to the Q value function, then the system state transfers to the next state $s_{t+1}$. With the continuous exploration of Q-learning, we use Equation (26) to update the Q value function:

(26) $$Q\left(s_{t},a_{t}\right)\leftarrow Q\left(s_{t},a_{t}\right)+\alpha \left[R_{t}+\gamma \max_{a\in A}Q\left(s_{t+1},a\right)-Q\left(s_{t},a_{t}\right)\right],$$

where α∈(0,1] is the learning rate.

Subsequently, the optimal action can be obtained through Equation (27). The system will randomly select an action with a certain probability to ensure that the algorithm has a certain degree of exploration. Finally, the optimal policy is obtained.

(27) $$a^{*}=\max_{a\in A}Q\left(s,a\right).$$

According to the above process, the fusion system obtains the optimal action by repeatedly calculating and updating the *Q* value. As a result, the BPAs in conflict are deleted, consistent BPAs are retained, which can realize the adaptive online information processing. After processing all the evidence, in this paper, the DCR is used to achieve MSIF. The proposed method is outlined in Algorithm 1.
**Algorithm 1** The proposed evidence combination algorithm.1:**Input:** BPAs from *m* sensors; state space *S*; action space *A*; discount factor γ; learning rate α; episode number *M*.2:**Initialization**Q(s,a) table.3:**for** each episode **do**4: **for** *t* = 1 to *m* **do**5:  Initialize state *S*;6:  Observe current state $s_{t}$, and choose an action $a_{t}$ (use ε−greedy policy);7:  Take action $a_{t}$, calculate the negation of BPA, calculate the uncertainty degrees of original BPA and its negation according to Equation (22), obtain the reward value $R_{t}$ according to Equation (23), then the system transfers to next state $s_{t+1}$;8:  Utilize Equation (26) to update Q function;9:  Calculate fusion results according to Equation (4);10:  $S\leftarrow s_{t+1}$ is the final state.11: **end for**12:**end for**13:**Output:** Multi-sensor information fusion result.

### 3.3. Decision Making Based on Correlation Coefficient

In this paper, a decision-making scheme based on the correlation coefficient is proposed as follows.

A BPA $\hat{m}$ whose mass is fully assigned to an element of FOD is called baseline BPA, i.e, $\hat{m}\left(A\right)=1$, for any A∈Θ. Then, we calculate the correlation coefficient between each baseline BPA and the BPA obtained by combination. The proposition corresponding to the maximum correlation coefficient is the decision result.

(28) $$\hat{X}=\max_{A_{i}\in \Theta \backslash \left\{\emptyset \right\}}r_{BPA}\left(m\left(\cdot \right),\hat{m}\left(\cdot \right)\right),$$

where $\hat{X}$ is the final decision result, and $r_{BPA}\left(\cdot \right)$ is the correlation coefficient.

## 4. Simulation Analysis and Application

To evaluate the effectiveness of the proposed multi-sensor information fusion decision-making method, numerical examples are provided.

### 4.1. Numerical Example

#### 4.1.1. Numerical Example 1

The example is adapted from [39]. In this example, there are five sensors simultaneously detecting a target. Assume FOD is Θ={A,B,C}, which indicates that the target is one among *A*, *B*, and *C*. BPAs obtained from the five sensors are $m_{1},m_{2},m_{3},m_{4},m_{5}$, respectively, as shown in Table 1.

The proposed method in this paper is used to perform multi-sensor information fusion for the provided BPAs shown in Table 1. The detailed simulation parameters are summarized in Table 2. The evidence processing results are shown in Table 3. From the table, by using the proposed method, BPAs $m_{1}$, $m_{3}$, $m_{4}$, and $m_{5}$ are retained, while $m_{2}$ is deleted because it is highly conflicting with other BPAs. During the process, we can obtain the values of the negation of BPA. The detailed negation of the BPA is summarized in Table 4.

In Table, $\bar{m_{1}}$ is the negation of $m_{1}$, ⊗ is the fusion. According to the negation of BPA in Table 4, we can obtain the uncertainty of the other side of the evidence, which effectively enhances the expression of the uncertainty of the evidence.

**Figure 2:**
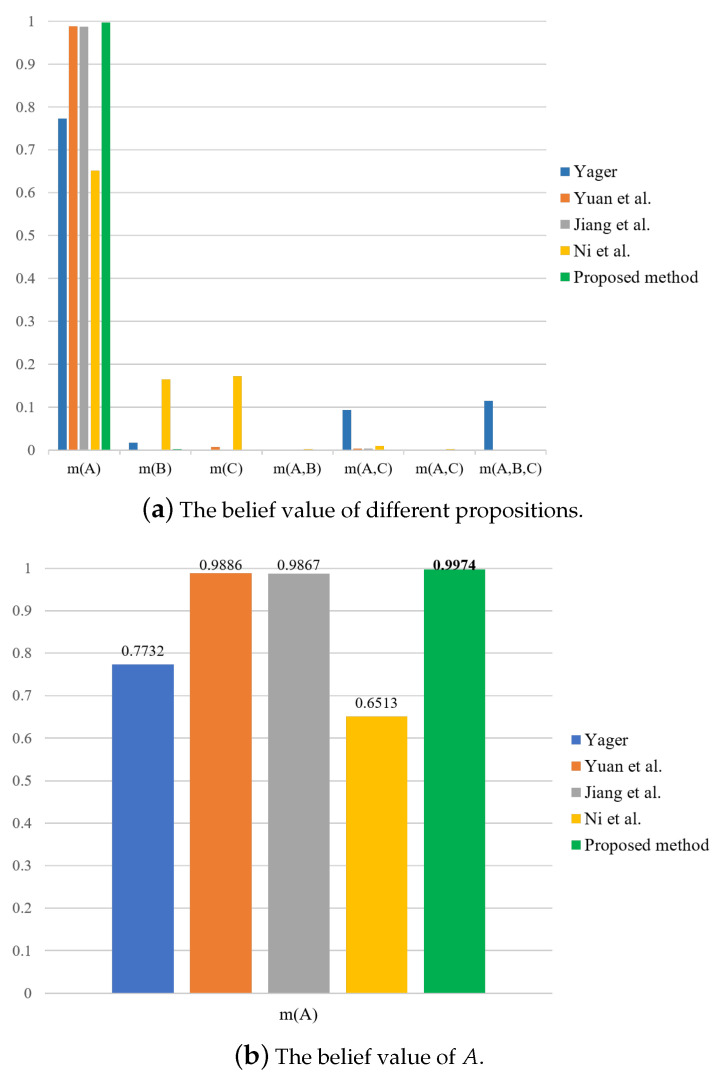
Comparison of fusion results for different methods in the numerical example.

Further, we compare the proposed method with four existing methods, including the methods from Yager [31], Yuan et al. [39], Jiang et al. [42], and Ni et al. [43]. The fusion results are shown in Table 5, which are also graphically shown in Figure 2. Then, by calculating the correlation coefficient of BPA *m* obtained by the combination with each baseline BPA, ${\hat{m}}_{A}\left(A\right)=1,\;{\hat{m}}_{B}\left(B\right)=1,\;{\hat{m}}_{C}\left(C\right)=1$, we have

(29) $$r_{BPA}\left({\hat{m}}_{A},m\right)=1,\;r_{BPA}\left({\hat{m}}_{B},m\right)=0.0026,\;r_{BPA}\left({\hat{m}}_{C},m\right)=\;0,$$

It can be seen that the proposition with the largest correlation coefficient is *A*, so the final decision result is *A*. Similarly, the decision results from other combination methods can be obtained as shown in Table 6. According to Table 5 and Table 6, by comparing these methods, it is found that the proposed method has the largest belief value on m(A), which is the most favorable for decision making.

#### 4.1.2. Numerical Example 2

Moreover, in order to fully demonstrate the importance of negative BPA in conflict management and multi-sensor information fusion, a numerical example is used to illustrate. The evidence of the numerical simulation example are shown in Table 7.

The evidence in Table 7 is used to explain in detail that the negation of BPA contributes to conflict management. Specifically, it can be divided into two cases. Case 1: only uses the uncertainty of the original BPA for conflict management. Case 2: uses the uncertainty of the original BPA and the uncertainty of the negative BPA for conflict management. By comparing the fusion result in the two cases, the importance of the negative BPA for conflict management and fusion results can be proved.

We can obtain the detailed negation of the BPA by calculating, which is summarized in Table 8. Further, we can obtain the uncertainty degrees in the calculation process, as shown follows.

It can be seen that if only the uncertainty of the original BPA is considered, $m_{s2}$ is deleted, which is because $E\left(m_{s1}⊗m_{s2}\right)=1.0020>E\left(m_{s1}\right)=0.8831$. Since $E\left(m_{s1}⊗m_{s3}\right)=0.6781<E\left(m_{s1}\right)=0.8813$, $m_{s3}$ is retained. We can know that, in this case, $m_{s1}$ and $m_{s3}$ are retained, $m_{s2}$ is deleted; therefore, the fusion result in this case is m(a)=0.8209,m(b)=0.1791.

If we not only consider the uncertainty of the original BPA, but also consider the uncertainty of the negative BPA. Sensor $m_{s2}$ is waiting to process in the first round of processing result, which is because $\left\{\begin{array}{c} E\left(m_{s1}⊗m_{s2}\right)=1.0020>E\left(m_{s1}\right)=0.8813\\ E\left(\bar{m_{s1}⊗m_{s2}}\right)=2.7261<E\left(\bar{m_{s1}}\right)=2.8330 \end{array}\right.$. Since $\left\{\begin{array}{c} E\left(m_{s1}⊗m_{s3}\right)=0.6781<E\left(m_{s1}\right)=0.8813\\ E\left(\bar{m_{s1}⊗m_{s3}}\right)=2.2631<E\left(\bar{m_{s1}}\right)=2.8330 \end{array}\right.$, $m_{s3}$ is retained. When all the sensor information is processed, $m_{s2}$ is processed for the second round. At this moment, we can find $\left\{\begin{array}{c} E\left(m_{s1}⊗m_{s3}⊗m_{s2}\right)=0.6283<E\left(m_{s1}⊗m_{s3}\right)=0.6781\\ E\left(\bar{m_{s1}⊗m_{s3}⊗m_{s2}}\right)=2.2133<E\left(\bar{m_{s1}⊗m_{s3}}\right)=2.2631 \end{array}\right.$, so in the second round of processing result, $m_{s2}$ is retained; therefore, the fusion result in this case is m(a)=0.8425,m(b)=0.1575.

From the above, we can see that if only the uncertainty of the original BPA is used for conflict management, the result may be single. When there are existing conflicts between one evidence and other evidence (i.e., in Table 7), this evidence will be deleted directly, which will result in the loss of part of the information. When the uncertainty of negative BPA is considered, the judgment conditions will be sufficient and the loss of information can be fully reduced. The above discussions demonstrate the effectiveness and reliability of negative BPA for conflict management. In addition, the fusion results show that the fusion result with the negation of BPA is more accurate. Thus, we consider that the negation of BPA can improve the belief value on m(a). It also demonstrates the effectiveness of the proposed method.

### 4.2. Application to Fault Diagnosis and Analysis

#### 4.2.1. Application to Fault Diagnosis

An application from [54] about fault diagnosis is examined herein. Assuming a motor rotor could have three different fault types, defined as, $F_{1}$, $F_{2}$, and $F_{3}$. The fault information is obtained through three sensors, under three different features, as shown in Table 9a–c. In Table 9, $m_{S1}$, $m_{S2}$, and $m_{S3}$ represent the evidence collected by the three sensors. In this paper, the true fault type of the motor rotor is $F_{2}$. By using the proposed method with the setting of parameters in Table 10, the evidence processing results are shown in Table 11. During the process, we can obtain the values of the negation of BPA, which are shown in Table 12.

We can know from Table 11 and Table 12, the BPAs for the application under feature 1, the processing result of sensor 3 in the first round is waiting to process, and the final round of processing result is deletion. It can be seen from the simulation results that the accuracy of the fusion result is improved when the evidence of sensor 3 is deleted, which indicates that the negation of BPA can improve the accuracy of the fusion result. The BPAs in the application under feature 2 and feature 3, which can provide a larger amount of information, and the conflict between BPAs is small, hence sensor 2 and sensor 3 are retained.

**Figure 3:**
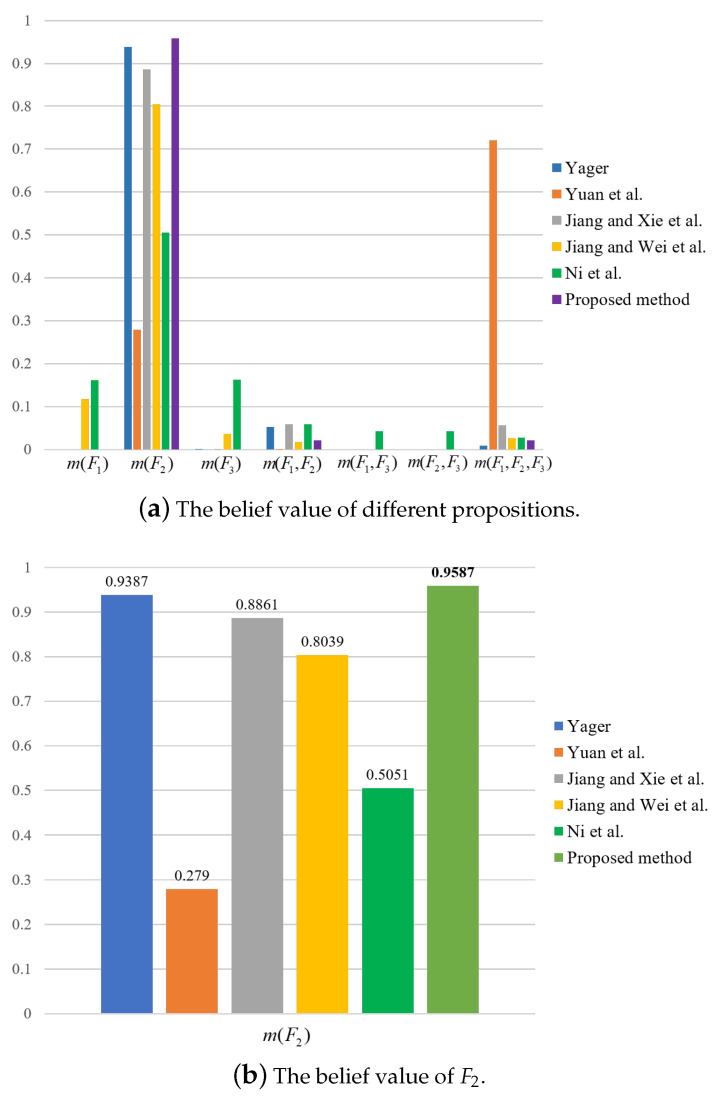
Comparison of fusion results for different methods under feature 1.

**Figure 4:**
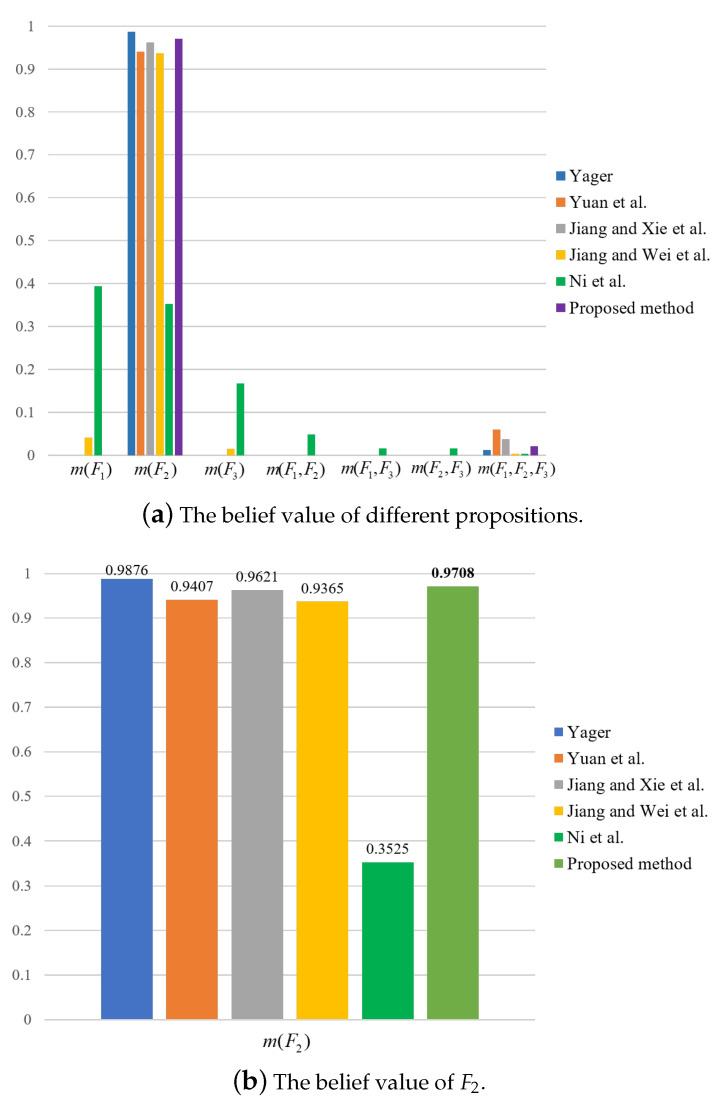
Comparison of fusion results for different methods under feature 2.

**Figure 5:**
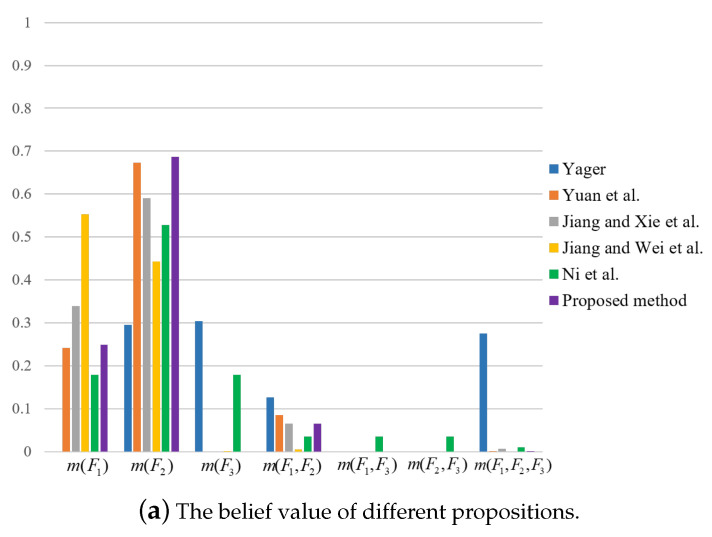

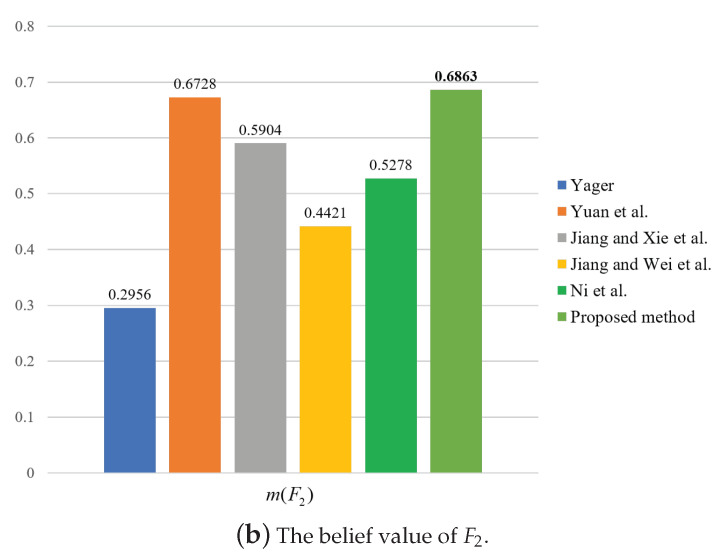
Comparison of fusion results for different methods under feature 3.

For the sake of comparison, results by the use of other methods are also obtained, as shown in Table 13 and Table 14 and Figure 3, Figure 4 and Figure 5. It can be seen from Table 13 and Table 14 that the proposed method has the highest mass or belief on the true fault type $F_{2}$ under each of the three features. This is because the proposed method can delete the conflicting evidence adaptively through RL, uncertainty degree of BPAs, and the negation of BPA, so as to avoid the impact of the conflicting evidence on the overall fusion accuracy. In addition, the proposed method can make full use of the sensor information to obtain the fusion results. By contrast, in the fusion result of Yager’s method $m(F_{3})$ is the largest under feature 3, which is inconsistent with the true fault type. As for Ni et al.’s method, the decision result is $F_{1}$ under feature 2, which is inconsistent with the true fault type. The other methods can identity the true fault type but the mass or belief of the result is lower than the proposed method.

In this paper, uncertainty of BPA and RL are combined to achieve multi-sensor information fusion. Thus, the analysis of the simulation results in this paper is enhanced from the perspective of uncertainty. Deng entropy and the entropy of Pal et al. [55,56] are used to measure the uncertainty of BPA, so as to judge its influence on the fusion result. The fusion results under two different entropies are consistent; however, the use of Deng entropy makes the convergence speed of the algorithm better than the entropy of Pal et al. The algorithm converges when the number of episodes is 55 and 58, respectively. Due to the small amount of information in this paper, there is little difference in convergence speed between different algorithms; however, this phenomenon also shows the importance of using Dun entropy to calculate BPA uncertainty.

#### 4.2.2. Robustness Analysis

Since the fusion result application cannot fully reflect the robustness of the proposed method, we focus on the analysis of the robustness in the application. Specifically, in order to fully reflect the robustness of the method in this paper when conflict is increasing, we adjust the evidence in application to fault diagnosis. When conflict is increasing, we calculate the fusion result of the proposed method. For the evidence in Table 9a, we first assign the belief value of $m(F_{2})$ in sensor 2 to $m(F_{1})$ at 0.05 intervals. Then, we assign the belief value of $m(F_{1},F_{2},F_{3})$ in sensor 3 to $m(F_{3})$ at 0.05 intervals. In addition, the evidence of sensor 1 remains unchanged. For the evidence in Table 9b, we first assign the belief value of $m(F_{2})$ in sensor 1 to $m(F_{1})$ at 0.05 intervals. Then, we assign the belief value of $m(F_{2})$ in sensor 2 to $m(F_{3})$ at 0.05 intervals. In addition, the evidence of sensor 3 remains unchanged. For the evidence in Table 9c, we first assign the belief value of $m(F_{1},F_{2})$ in sensor 2 to $m(F_{1})$ at 0.03 intervals. Then, we assign the belief value of $m(F_{1},F_{2})$ in sensor 3 to $m(F_{3})$ at 0.03 intervals. In addition, the evidence of sensor 1 remains unchanged. According to the above discussion, the adjusted BPAs are shown in Table 15, Table 16 and Table 17.

In Table 15, Table 16 and Table 17, we adopt the conflict calculation method based on correlation coefficient proposed by the Jiang [50] to calculate the degree of conflict. The degree of conflict is defined as:

(30) $$C_{ij}=1-r_{BPA}\left(m_{i},m_{j}\right)=1-\frac{c(m_{i},m_{j})}{\sqrt{c\left(m_{i},m_{i}\right)\times c\left(m_{j},m_{j}\right)}},$$

where $C_{ij}$ represent the degree of conflict, $m_{i}$ and $m_{j}$ denote the evidence of the *i*-th and *j*-th sensors, respectively, and $c\left(m_{i},m_{j}\right)=\sum_{p=1}^{2^{N}}\sum_{q=1}^{2^{N}}m_{i}\left(A_{p}\right)m_{j}\left(A_{q}\right)\frac{\left|A_{p}\cap A_{q}\right|}{\left|A_{p}\cup A_{q}\right|}$ is the degree of correlation.

From Table 15, it can be seen that in the evidence under feature 1 after adjustment, the conflict between sensor 1 and sensor 2 has been increasing. The conflict degree between sensors 1 and 3 first decreases and then increases. The conflict degree between sensors 2 and 3 first decreases and then increases; however, it can be seen from the whole that the degree of conflict between adjusted evidence is gradually increasing. From Table 16, it can be seen that in the evidence under feature 2 after adjustment, the degree of conflict between sensor 1, sensor 2, and sensor 3 has been increasing, and it is obvious. In the evidence under feature 3, the belief value of the single subset is relatively small, and the distribution of belief value is relatively uniform. For these reasons, we adjust the evidence to a relatively small extent. Whereas, in Table 17, we can know that the conflicts between the evidence is also changing significantly.

According to the evidence in Table 15, Table 16 and Table 17, the fusion results under different cases can be obtained by using the proposed method in this paper, as shown in Table 18.

From Table 16, we can know that, with the conflict between evidence increasing, the proposed method in this paper can still obtain accurate fusion results; however, the belief value on $m(F_{2})$ decreases as the conflict increases. This is mainly shown as follows. In the evidence under feature 1, the belief value on $m(F_{2})$ is reduced from 0.9587 to 0.9129. In the evidence under feature 2, the belief value on $m(F_{2})$ is reduced from 0.9708 to 0.8875. In the evidence under feature 3, the belief value on $m(F_{2})$ is reduced from 0.6863 to 0.6108.

As can be seen from the simulation results, the proposed method can obtain effective fusion results; however, there are still some limitations in the fusion results. Specifically, the belief values are particularly concentrated, mainly on $m(F_{2})$ and $m(F_{1},F_{2},F_{3})$. In this case, if BPAs fluctuates greatly, the conflict between evidence will increase. Then the fusion results made by the proposed method will fluctuate greatly; however, the simulation results show that the proposed method can also obtain effective fusion results when conflict is increasing. Thus, the robustness of the proposed method can be verified.

## 5. Conclusions

In this paper, we have investigated the multi-sensor online fusion problem, and proposed a novel method on the basis of the uncertainty of BPA and RL. Specially, the proposed method has measured the uncertain degrees of original BPA and its negation by the use of Deng entropy. Then, the two uncertain degrees and RL have been combined to achieve the online conflicting management. The above process has the advantages of making full use of the information and reducing the loss of information. On the basis of selected BPAs, DCR has been used for evidence combination. Finally, a decision scheme based on the correlation coefficient has been adopted to obtain the decision-making result. Simulation results of numerical example and application have demonstrated the effectiveness of the proposed method. In a future study, the application of the proposed method will be further investigated.

In addition to those problems listed above, there are many research issues beckoning for further investigation. In this paper, we focus on the multi-sensor fusion decision-making problem with a small amount of information, and ignore how to quickly and accurately obtain the fusion result when the amount of sensor information is significant. Nevertheless, the proposed method proposed provides an idea for the application of artificial intelligence in multi-sensor fusion. As a future work, we plan to use neural networks and RL, and combine them with our proposed algorithm for an actual fusion decision-making system.

## Figures and Tables

**Table 1:** BPAs in the numerical example 1.

BPA	m(A)	m(B)	m(C)	m(A,C)
Sensor 1: $m_{1}$	0.41	0.29	0.30	0
Sensor 2: $m_{2}$	0	0.90	0.10	0
Sensor 3: $m_{3}$	0.58	0.07	0	0.35
Sensor 4: $m_{4}$	0.55	0.10	0	0.35
Sensor 5: $m_{5}$	0.60	0.10	0	0.30

**Table 2:** Simulation parameters for the numerical example 1.

Parameter	Value
Discount factor (γ)	0.9
Learning rate (α)	0.1
Episode number (*M*)	100

**Table 3:** Results of online processing of BPAs for the numerical example 1.

**BPA**	Sensor 1: $m_{1}$	Sensors 2: $m_{2}$	Sensor 3: $m_{3}$	Sensor 4: $m_{4}$	Sensor 5: $m_{5}$
**Processing result**	Retain	Delete	Retain	Retain	Retain

**Table 4:** The negation of the BPAs in the numerical example 1.

The Negation of BPA	m(B,C)	m(A,C)	m(A,B,C)
$\bar{m_{1}}$	0.41	0.29	0.30
$\bar{m_{1}⊗m_{2}}$	0	0.8969	0.1031
$\bar{m_{1}⊗m_{3}}$	0.9213	0.0787	0
$\bar{m_{1}⊗m_{3}⊗m_{4}}$	0.9847	0.0153	0
$\bar{m_{1}⊗m_{3}⊗m_{4}⊗m_{5}}$	0.9974	0.0026	0

**Table 5:** Fusion results of different methods for the numerical example 1.

Methods	m(A)	m(B)	m(C)	m(A,B)	m(A,C)	m(B,C)	m(A,B,C)
Yager [31]	0.7732	0.0167	0.0011	0	0.0938	0	0.1152
Yuan et al. [39]	0.9886	0.0002	0.0072	0	0.0039	0	0
Jiang et al. [42]	0.9867	0.0008	0	0	0.0036	0	0
Ni et al. [43]	0.6513	0.1648	0.1730	0.0016	0.0096	0.0016	0
Proposed method	0.9974	0.0026	0	0	0	0	0

**Table 6:** Decision-making results of different methods for the numerical example 1.

Methods	$r_{\mathrm{BPA}}\left({\hat{m}}_{A},m\right)$	$r_{\mathrm{BPA}}\left({\hat{m}}_{B},m\right)$	$r_{\mathrm{BPA}}\left({\hat{m}}_{C},m\right)$	Decision-Making Result
Yager [31]	0.9750	0.0532	0.0716	*A*
Yuan et al. [39]	1	0.0002	0.0086	*A*
Jiang et al. [42]	1	0.0008	0.0012	*A*
Ni et al. [43]	0.9378	0.2375	0.2530	*A*
Proposed method	1	0.0026	0	*A*

**Table 7:** BPAs in numerical example 2.

BPA	m(a)	m(b)	m(c)	m(a,b)	m(b,c)
$m_{s1}$	0.7	0	0	0.3	0
$m_{s2}$	0.4	0	0	0.3	0.3
$m_{s3}$	0.55	0.2	0.05	0	0.2

**Table 8:** The negation of the BPAs in numerical example 2.

The Negation of BPA	m(b,c)	m(a,c)	m(a,b)	m(a,b,c)
$\bar{m_{s1}}$	0.7	0	0	0.30
$\bar{m_{s1}⊗m_{s2}}$	0.7722	0.1139	0	0.1139
$\bar{m_{s1}⊗m_{s3}}$	0.8209	0.1791	0	0
$\bar{m_{s1}⊗m_{s2}⊗m_{s3}}$	0.8425	0.1575	0	0
$\begin{array}{c} E\left(m_{s1}\right)=0.8813,E\left(\bar{m_{s1}}\right)=2.8330\\ E\left(m_{s1}⊗m_{s2}\right)=1.0020,E\left(\bar{m_{s1}⊗m_{s2}}\right)=2.7261\\ E\left(m_{s1}⊗m_{s3}\right)=0.6781,E\left(\bar{m_{s1}⊗m_{s3}}\right)=2.2631\\ E\left(m_{s1}⊗m_{s3}⊗m_{s2}\right)=0.6283,E\left(\bar{m_{s1}⊗m_{s3}⊗m_{s2}}\right)=2.2133 \end{array}$				

**Table 9:** BPAs for the application.

(**a**) BPAs for the application under feature 1.				
**BPA**	$m(F_{2})$	$m(F_{3})$	$m(F_{1},F_{2})$	$m(F_{1},F_{2},F_{3})$
Sensor 1: $m_{S1}$	0.8176	0.0003	0.1553	0.0268
Sensor 2: $m_{S2}$	0.5658	0.0009	0.0646	0.3687
Sensor 3: $m_{S3}$	0.2403	0.0004	0.0141	0.7452
(**b**) BPAs for the application under feature 2.				
	**BPA**	$m(F_{2})$	$m(F_{1},F_{2},F_{3})$	
	Sensor 1: $m_{S1}$	0.6229	0.3771	
	Sensor 2: $m_{S2}$	0.7660	0.2340	
	Sensor 3: $m_{S3}$	0.8598	0.1402	
(**c**) BPAs for the application under feature 3.				
**BPA**	$m(F_{1})$	$m(F_{2})$	$m(F_{1},F_{2})$	$m(F_{1},F_{2},F_{3})$
Sensor 1: $m_{S1}$	0.3666	0.4563	0.1185	0.0586
Sensor 2: $m_{S2}$	0.2793	0.4151	0.2652	0.0404
Sensor 3: $m_{S3}$	0.2897	0.4331	0.2470	0.0302

**Table 10:** Simulation parameters for the application.

Parameter	Value
Discount factor (γ)	0.9
Learning rate (α)	0.1
Episode number (*M*)	80

**Table 11:** Results of online processing of BPAs for the application under different features.

BPA		The First Round of Processing Results	The Final Round of Processing Results
Feature 1	Sensor 1: $m_{S1}$	Retain	Retain
	Sensor 2: $m_{S2}$	Retain	Retain
	Sensor 3: $m_{S3}$	Waiting to Process	Delete
Feature 2	Sensor 1: $m_{S1}$	Retain	Retain
	Sensor 2: $m_{S2}$	Retain	Retain
	Sensor 3: $m_{S3}$	Retain	Retain
Feature 3	Sensor 1: $m_{S1}$	Retain	Retain
	Sensor 2: $m_{S2}$	Retain	Retain
	Sensor 3: $m_{S3}$	Retain	Retain

**Table 12:** The negation of the BPAs for the application.

(**a**) The negation of the BPAs for the application under feature 1.			
**The Negation of BPA**	$m(F_{1},F_{3})$	$m(F_{1},F_{2})$	$m(F_{1},F_{2},F_{3})$
$\bar{m_{S1}}$	0.8176	0.0003	0.1821
$\bar{m_{S1}⊗m_{S2}}$ $m_{S2}$	0.9587	0	0.0432
$\bar{m_{S1}⊗m_{S2}⊗m_{S3}}$ $m_{S3}$	0.9368	0	0.0632
(**b**) The negation of the BPAs for the application under feature 2.			
**The Negation of BPA**	$m(F_{1},F_{3})$	$m(F_{1},F_{2},F_{3})$	
$\bar{m_{S1}}$	0.6229	0.3771	
$\bar{m_{S1}⊗m_{S2}}$	0.8440	0.1562	
$\bar{m_{S1}⊗m_{S2}⊗m_{S3}}$ $m_{S3}$	0.9708	0.0292	
(**c**) The negation of the BPAs for the application under feature 3.			
**The Negation of BPA**	$m(F_{2},F_{3})$	$m(F_{1},F_{3})$	$m(F_{1},F_{2},F_{3})$
$\bar{m_{S1}}$	0.3666	0.4563	0.1771
$\bar{m_{S1}⊗m_{S2}}$	0.3145	0.5817	0.1038
$\bar{m_{S1}⊗m_{S2}⊗m_{S3}}$	0.2482	0.6863	0.0655

**Table 13:** Fusion results of different methods for the application.

(**a**) Fusion results of different methods for the application under feature 1.							
**Methods**	$m(F_{1})$	$m(F_{2})$	$m(F_{3})$	$m(F_{1},F_{2})$	$m(F_{1},F_{3})$	$m(F_{2},F_{3})$	$m(F_{1},F_{2},F_{3})$
Yager [31]	0	0.9387	0.0001	0.0526	0	0	0.0086
Yuan et al. [39]	0	0.2790	0	0.0003	0	0	0.7207
Jiang and Xie et al. [54]	0	0.8861	0.0002	0.0582	0	0	0.0555
Jiang and Wei et al. [42]	0.1178	0.8039	0.0356	0.0170	0	0	0.0257
Ni et al. [43]	0.1616	0.5051	0.1619	0.0587	0.0425	0.0425	0.0276
Proposed method	0	0.9587	0	0.0208	0	0	0.0205
(**b**) Fusion results of different methods for the application under feature 2.							
**Methods**	$m(F_{1})$	$m(F_{2})$	$m(F_{3})$	$m(F_{1},F_{2})$	$m(F_{1},F_{3})$	$m(F_{2},F_{3})$	$m(F_{1},F_{2},F_{3})$
Yager [31]	0	0.9876	0	0	0	0	0.0124
Yuan et al. [39]	0	0.9407	0	0	0	0	0.0593
Jiang and Xie et al. [54]	0	0.9621	0	0	0	0	0.0371
Jiang and Wei et al. [42]	0.0461	0.9365	0.0144	0	0	0	0.0030
Ni et al. [43]	0.3938	0.3525	0.1679	0.0487	0.0162	0.0162	0.0030
Proposed method	0	0.9708	0	0	0	0	0.0292
(**c**) Fusion results of different methods for the application under feature 3.							
**Methods**	$m(F_{1})$	$m(F_{2})$	$m(F_{3})$	$m(F_{1},F_{2})$	$m(F_{1},F_{3})$	$m(F_{2},F_{3})$	$m(F_{1},F_{2},F_{3})$
Yager [31]	0	0.2956	0.3034	0.1260	0	0	0.2750
Yuan et al. [39]	0.2414	0.6728	0	0.0852	0	0	0.0006
Jiang and Xie et al. [54]	0.3384	0.5904	0	0.0651	0	0	0.0061
Jiang and Wei et al. [42]	0.4421	0.5528	0.0005	0.0046	0	0	0
Ni et al. [43]	0.1787	0.5278	0.1787	0.0348	0.0348	0.0348	0.0097
Proposed method	0.2482	0.6863	0	0.0649	0	0	0.0006

**Table 14:** Decision-making results of different methods for the application.

(**a**) The correlation value under feature 1.				
**Methods**	$r_{\mathrm{BPA}}\left({\hat{m}}_{F_{1}},m_{S1}\right)$	$r_{\mathrm{BPA}}\left({\hat{m}}_{F_{2}},m_{S2}\right)$	$r_{\mathrm{BPA}}\left({\hat{m}}_{F_{3}},m_{S3}\right)$	**Decision-Making Result**
Yager [31]	0.0205	0.9983	0.0023	$F_{2}$
Yuan et al. [39]	0.2158	0.5497	0.2156	$F_{2}$
Jiang and Xie et al. [54]	0.0360	0.9940	0.0152	$F_{2}$
Jiang and Wei et al. [42]	0.1569	0.9854	0.0507	$F_{2}$
Ni et al. [43]	0.3225	0.8700	0.3141	$F_{2}$
Proposed method	0.0124	**0.9993**	0.0053	$F_{2}$
(**b**) The correlation value under feature 2.				
**Methods**	$r_{\mathrm{BPA}}\left({\hat{m}}_{F_{1}},m_{S1}\right)$	$r_{\mathrm{BPA}}\left({\hat{m}}_{F_{2}},m_{S2}\right)$	$r_{\mathrm{BPA}}\left({\hat{m}}_{F_{3}},m_{S3}\right)$	**Decision-Making Result**
Yager [31]	0.0031	0.9999	0.0031	$F_{2}$
Yuan et al. [39]	0.0155	0.9982	0.0155	$F_{2}$
Jiang and Xie et al. [54]	0.0095	0.9993	0.0095	$F_{2}$
Jiang and Wei et al. [42]	0.0499	0.9986	0.0161	$F_{2}$
Ni et al. [43]	0.7036	0.6337	0.3034	$F_{1}$
Proposed method	0	**0.9996**	0.0099	$F_{2}$
(**c**) The correlation value under feature 3.				
**Methods**	$r_{\mathrm{BPA}}\left({\hat{m}}_{F_{1}},m_{S1}\right)$	$r_{\mathrm{BPA}}\left({\hat{m}}_{F_{2}},m_{S2}\right)$	$r_{\mathrm{BPA}}\left({\hat{m}}_{F_{3}},m_{S3}\right)$	**Decision-Making Result**
Yager [31]	0.1689	0.5675	0.6196	$F_{3}$
Yuan et al. [39]	0.3574	0.9286	0.0002	$F_{2}$
Jiang and Xie et al. [54]	0.5058	0.8583	0.0021	$F_{2}$
Jiang and Wei et al. [42]	0.6248	0.7807	0.0007	$F_{2}$
Ni et al. [43]	0.3244	0.8787	0.3244	$F_{2}$
Proposed method	0.3552	**0.9317**	0.0002	$F_{2}$

**Table 15:** BPAs of different conflicts for the application under feature 1.

No.	BPA	$m(F_{1})$	$m(F_{2})$	$m(F_{3})$	$m(F_{1},F_{2})$	$m(F_{1},F_{2},F_{3})$	Conflict Degree
1	Sensor 1: $m_{S1}$	0	0.8176	0.0003	0.1553	0.0268	$\begin{array}{c} C_{12}=0.0738\\ C_{13}=0.3714\\ C_{23}=0.1217 \end{array}$
	Sensor 2: $m_{S2\_1}$	0	0.5658	0.0009	0.0646	0.3687	
	Sensor 3: $m_{S3\_1}$	0	0.2403	0.0004	0.0141	0.7452	
2	Sensor 1: $m_{S1}$	0	0.8176	0.0003	0.1553	0.0268	$\begin{array}{c} C_{12}=0.0958\\ C_{13}=0.3640\\ C_{23}=0.1012 \end{array}$
	Sensor 2: $m_{S2\_2}$	0	0.5158	0.0509	0.0646	0.3687	
	Sensor 3: $m_{S3\_2}$	0.05	0.2403	0.0004	0.0141	0.6952	
3	Sensor 1: $m_{S1}$	0	0.8176	0.0003	0.1553	0.0268	$\begin{array}{c} C_{12}=0.1262\\ C_{13}=0.3586\\ C_{23}=0.0871 \end{array}$
	Sensor 2: $m_{S2\_3}$	0	0.4658	0.1009	0.0646	0.3687	
	Sensor 3: $m_{S3\_3}$	0.1	0.2403	0.0004	0.0141	0.6452	
4	Sensor 1: $m_{S1}$	0	0.8176	0.0003	0.1553	0.0268	$\begin{array}{c} C_{12}=0.1663\\ C_{13}=0.3559\\ C_{23}=0.0860 \end{array}$
	Sensor 2: $m_{S2\_4}$	0	0.4158	0.1509	0.0646	0.3687	
	Sensor 3: $m_{S3\_4}$	0.15	0.2403	0.0004	0.0141	0.5952	
5	Sensor 1: $m_{S1}$	0	0.8176	0.0003	0.1553	0.0268	$\begin{array}{c} C_{12}=0.2166\\ C_{13}=0.3565\\ C_{23}=0.1009 \end{array}$
	Sensor 2: $m_{S2\_5}$	0	0.3658	0.2009	0.0646	0.3687	
	Sensor 3: $m_{S3\_5}$	0.2	0.2403	0.0004	0.0141	0.5452	
6	Sensor 1: $m_{S1}$	0	0.8176	0.0003	0.1553	0.0268	$\begin{array}{c} C_{12}=0.2771\\ C_{13}=0.3613\\ C_{23}=0.1338 \end{array}$
	Sensor 2: $m_{S2\_6}$	0	0.3158	0.2509	0.0646	0.3687	
	Sensor 3: $m_{S3\_6}$	0.25	0.2403	0.0004	0.0141	0.4952	
7	Sensor 1: $m_{S1}$	0	0.8176	0.0003	0.1553	0.0268	$\begin{array}{c} C_{12}=0.3466\\ C_{13}=0.3707\\ C_{23}=0.1852 \end{array}$
	Sensor 2: $m_{S2\_7}$	0	0.2658	0.3009	0.0646	0.3687	
	Sensor 3: $m_{S3\_7}$	0.3	0.2403	0.0004	0.0141	0.4452	
8	Sensor 1: $m_{S1}$	0	0.8176	0.0003	0.1553	0.0268	$\begin{array}{c} C_{12}=0.4231\\ C_{13}=0.3852\\ C_{23}=0.2535 \end{array}$
	Sensor 2: $m_{S2\_8}$	0	0.2158	0.3509	0.0646	0.3687	
	Sensor 3: $m_{S3\_8}$	0.35	0.2403	0.0004	0.0141	0.3952	

**Table 16:** BPAs of different conflicts for the application under feature 2.

No.	BPA	$m(F_{1})$	$m(F_{2})$	$m(F_{3})$	$m(F_{1},F_{2},F_{3})$	Conflict Degree
1	Sensor 1: $m_{S1\_1}$	0	0.6229	0	0.3771	$\begin{array}{c} C_{12}=0.0205\\ C_{13}=0.0509\\ C_{23}=0.0069 \end{array}$
	Sensor 2: $m_{S2\_1}$	0	0.7660	0	0.2340	
	Sensor 3: $m_{S3}$	0	0.8598	0	0.1402	
2	Sensor 1: $m_{S1\_2}$	0.05	0.5729	0	0.3771	$\begin{array}{c} C_{12}=0.0271\\ C_{13}=0.0677\\ C_{23}=0.0129 \end{array}$
	Sensor 2: $m_{S2\_2}$	0	0.7160	0.05	0.2340	
	Sensor 3: $m_{S3}$	0	0.8598	0	0.1402	
3	Sensor 1: $m_{S1\_3}$	0.1	0.5229	0	0.3771	$\begin{array}{c} C_{12}=0.0433\\ C_{13}=0.0924\\ C_{23}=0.0247 \end{array}$
	Sensor 2: $m_{S2\_3}$	0	0.6660	0.1	0.2340	
	Sensor 3: $m_{S3}$	0	0.8598	0	0.1402	
4	Sensor 1: $m_{S1\_4}$	0.15	0.4729	0	0.3771	$\begin{array}{c} C_{12}=0.0713\\ C_{13}=0.1263\\ C_{23}=0.0439 \end{array}$
	Sensor 2: $m_{S2\_4}$	0	0.6160	0.15	0.2340	
	Sensor 3: $m_{S3}$	0	0.8598	0	0.1402	
5	Sensor 1: $m_{S1\_5}$	0.2	0.4229	0	0.3771	$\begin{array}{c} C_{12}=0.1123\\ C_{13}=0.1704\\ C_{23}=0.0722 \end{array}$
	Sensor 2: $m_{S2\_5}$	0	0.5660	0.2	0.2340	
	Sensor 3: $m_{S3}$	0	0.8598	0	0.1402	
6	Sensor 1: $m_{S1\_6}$	0.25	0.3729	0	0.3771	$\begin{array}{c} C_{12}=0.1666\\ C_{13}=0.2250\\ C_{23}=0.1108 \end{array}$
	Sensor 2: $m_{S2\_6}$	0	0.5160	0.25	0.2340	
	Sensor 3: $m_{S3}$	0	0.8598	0	0.1402	
7	Sensor 1: $m_{S1\_7}$	0.3	0.3229	0	0.3771	$\begin{array}{c} C_{12}=0.2327\\ C_{13}=0.2893\\ C_{23}=0.1604 \end{array}$
	Sensor 2: $m_{S2\_7}$	0	0.4660	0.3	0.2340	
	Sensor 3: $m_{S3}$	0	0.8598	0	0.1402	
8	Sensor 1: $m_{S1\_8}$	0.35	0.2729	0	0.3771	$\begin{array}{c} C_{12}=0.3074\\ C_{13}=0.3617\\ C_{23}=0.2210 \end{array}$
	Sensor 2: $m_{S2\_8}$	0	0.4160	0.35	0.2340	
	Sensor 3: $m_{S3}$	0	0.8598	0	0.1402	

**Table 17:** BPAs of different conflicts for the application under feature 3.

No.	BPA	$m(F_{1})$	$m(F_{2})$	$m(F_{3})$	$m(F_{1},F_{2})$	$m(F_{1},F_{2},F_{3})$	Conflict Degree
1	Sensor 1: $m_{S1}$	0.3666	0.4563	0	0.1185	0.0586	$\begin{array}{c} C_{12}=0.0167\\ C_{13}=0.0118\\ C_{23}=0.0007 \end{array}$
	Sensor 2: $m_{S2\_1}$	0.2793	0.4151	0	0.2652	0.0404	
	Sensor 3: $m_{S3\_1}$	0.2897	0.4331	0	0.2470	0.0302	
2	Sensor 1: $m_{S1}$	0.3666	0.4563	0	0.1185	0.0586	$\begin{array}{c} C_{12}=0.0096\\ C_{13}=0.0088\\ C_{23}=0.0021 \end{array}$
	Sensor 2: $m_{S2\_2}$	0.3093	0.4151	0	0.2352	0.0404	
	Sensor 3: $m_{S3\_2}$	0.2897	0.4331	0.03	0.2170	0.0302	
3	Sensor 1: $m_{S1}$	0.3666	0.4563	0	0.1185	0.0586	$\begin{array}{c} C_{12}=0.0050\\ C_{13}=0.0089\\ C_{23}=0.0073 \end{array}$
	Sensor 2: $m_{S2\_3}$	0.3393	0.4151	0	0.2052	0.0404	
	Sensor 3: $m_{S3\_3}$	0.2897	0.4331	0.06	0.1870	0.0302	
4	Sensor 1: $m_{S1}$	0.3666	0.4563	0	0.1185	0.0586	$\begin{array}{c} C_{12}=0.0028\\ C_{13}=0.0125\\ C_{23}=0.0164 \end{array}$
	Sensor 2:$m_{S2\_4}$	0.3693	0.4151	0	0.1752	0.0404	
	Sensor 3: $m_{S3\_4}$	0.2897	0.4331	0.09	0.1570	0.0302	
5	Sensor 1: $m_{S1}$	0.3666	0.4563	0	0.1185	0.0586	$\begin{array}{c} C_{12}=0.0033\\ C_{13}=0.0203\\ C_{23}=0.0298 \end{array}$
	Sensor 2: $m_{S2\_5}$	0.3993	0.4151	0	0.1452	0.0404	
	Sensor 3: $m_{S3\_5}$	0.2897	0.4331	0.12	0.1270	0.0302	
6	Sensor 1: $m_{S1}$	0.3666	0.4563	0	0.1185	0.0586	$\begin{array}{c} C_{12}=0.0063\\ C_{13}=0.0326\\ C_{23}=0.0477 \end{array}$
	Sensor 2: $m_{S2\_6}$	0.4293	0.4151	0	0.1152	0.0404	
	Sensor 3: $m_{S3\_6}$	0.2897	0.4331	0.15	0.0970	0.0302	
7	Sensor 1: $m_{S1}$	0.3666	0.4563	0	0.1185	0.0586	$\begin{array}{c} C_{12}=0.0119\\ C_{13}=0.0499\\ C_{23}=0.0698 \end{array}$
	Sensor 2: $m_{S2\_7}$	0.4593	0.4151	0	0.0852	0.0404	
	Sensor 3: $m_{S3\_7}$	0.2897	0.4331	0.18	0.0670	0.0302	
8	Sensor 1: $m_{S1}$	0.3666	0.4563	0	0.1185	0.0586	$\begin{array}{c} C_{12}=0.0199\\ C_{13}=0.0723\\ C_{23}=0.0961 \end{array}$
	Sensor 2: $m_{S2\_8}$	0.4893	0.4151	0	0.0552	0.0404	
	Sensor 3: $m_{S3\_8}$	0.2897	0.4331	0.21	0.0370	0.0302	

**Table 18:** Fusion results for the application.

(**a**) Fusion results under feature 1.							
**No.**	$m(F_{1})$	$m(F_{2})$	$m(F_{3})$	$m(F_{1},F_{2})$	$m(F_{1},F_{3})$	$m(F_{2},F_{3})$	$m(F_{1},F_{2},F_{3})$
1	0	0.9587	0	0.0208	0	0	0.0205
2	0	0.9549	0	0.0227	0	0	0.0224
3	0	0.9502	0	0.0251	0	0	0.0247
4	0	0.9445	0	0.0279	0	0	0.0276
5	0	0.9374	0.0002	0.0314	0	0	0.0314
6	0	0.9281	0.0003	0.0361	0	0	0.0355
7	0	0.9200	0	0.0025	0	0	0.0775
8	0	0.9129	0	0.0030	0	0	0.0841
(**b**) Fusion results under feature 2.							
**No.**	$m(F_{1})$	$m(F_{2})$	$m(F_{3})$	$m(F_{1},F_{2})$	$m(F_{1},F_{3})$	$m(F_{2},F_{3})$	$m(F_{1},F_{2},F_{3})$
1	0	0.9708	0	0	0	0	0.0292
2	0	0.9661	0	0	0	0	0.0339
3	0	0.9603	0	0	0	0	0.0397
4	0	0.9529	0	0	0	0	0.0471
5	0	0.9433	0	0	0	0	0.0567
6	0	0.9304	0	0	0	0	0.0696
7	0	0.9127	0	0	0	0	0.0873
8	0	0.8875	0	0	0	0	0.1125
(**c**) Fusion results under feature 3.							
**No.**	$m(F_{1})$	$m(F_{2})$	$m(F_{3})$	$m(F_{1},F_{2})$	$m(F_{1},F_{3})$	$m(F_{2},F_{3})$	$m(F_{1},F_{2},F_{3})$
1	0.2482	0.6863	0	0.0649	0	0	0.0006
2	0.2715	0.6780	0	0.0500	0	0	0.0005
3	0.2837	0.6686	0	0.0371	0	0	0.0006
4	0.3148	0.6585	0	0.0262	0	0	0.0005
5	0.3347	0.6475	0	0.0172	0	0	0.0006
6	0.3534	0.6358	0	0.0103	0	0	0.0005
7	0.3708	0.6235	0	0.0051	0	0	0.0006
8	0.3869	0.6108	0	0.0018	0	0	0.0005

## Data Availability

Not applicable.
